# Portal vein embolization *versus* dual vein embolization for management of the future liver remnant in patients undergoing major hepatectomy: meta-analysis

**DOI:** 10.1093/bjsopen/zrac131

**Published:** 2022-11-18

**Authors:** Richard J Bell, Abdul R Hakeem, Sanjay Pandanaboyana, Brian R Davidson, Raj K Prasad, Bobby V M Dasari

**Affiliations:** Department of Hepatobiliary and Transplant Surgery, St James’s University Hospital, Leeds, UK; Department of Hepatobiliary and Transplant Surgery, St James’s University Hospital, Leeds, UK; Department of Hepato-Pancreato-Biliary (HPB) and Transplant Surgery, Freeman Hospital, Newcastle upon Tyne, UK; Department of Hepato-Pancreato-Biliary (HPB) and Transplant Surgery, Royal Free Hospital, London, UK; Department of Hepatobiliary and Transplant Surgery, St James’s University Hospital, Leeds, UK; Department of Hepato-Pancreato-Biliary (HPB) and Transplant Surgery, University Hospital Birmingham, Birmingham, UK; School of Medicine, University of Birmingham, Birmingham, UK

## Abstract

**Background:**

This meta-analysis aimed to compare progression to surgery, extent of liver hypertrophy, and postoperative outcomes in patients planned for major hepatectomy following either portal vein embolization (PVE) or dual vein embolization (DVE) for management of an inadequate future liver remnant (FLR).

**Methods:**

An electronic search was performed of MEDLINE, Embase, and PubMed databases using both medical subject headings (MeSH) and truncated word searches. Articles comparing PVE with DVE up to January 2022 were included. Articles comparing sequential DVE were excluded. ORs, risk ratios, and mean difference (MD) were calculated using fixed and random-effects models for meta-analysis.

**Results:**

Eight retrospective studies including 523 patients were included in the study. Baseline characteristics between the groups, specifically, age, sex, BMI, indication for resection, and baseline FLR (ml and per cent) were comparable. The percentage increase in hypertrophy was larger in the DVE group, 66 per cent in the DVE group *versus* 27 per cent in the PVE group, MD 39.07 (9.09, 69.05) (*P* = 0.010). Significantly fewer patients failed to progress to surgery in the DVE group than the PVE group, 13 per cent *versus* 25 per cent respectively OR 0.53 (0.31, 0.90) (*P* = 0.020). Rates of post-hepatectomy liver failure 13 per cent *versus* 22 per cent (*P* = 0.130) and major complications 20 per cent *versus* 28 per cent (Clavien–Dindo more than IIIa) (*P* = 0.280) were lower. Perioperative mortality was lower with DVE, 1 per cent *versus* 10 per cent (*P* = 0.010)

**Conclusion:**

DVE seems to produce a greater degree of hypertrophy of the FLR than PVE alone which translates into more patients progressing to surgery. Higher quality studies are needed to confirm these results.

## Teaser

DVE seems to produce a greater hypertrophy than PVE alone. This translates into more patients progressing to surgery with lower post-hepatectomy liver failure and mortality. Higher quality studies are needed to confirm these findings.

## Introduction

Colorectal cancer is the third most frequent cause of cancer-related death with up to 30 per cent diagnosed with metastatic spread to the liver. About 25 per cent of these patients will have potentially resectable disease and surgical resection represents the main curative option. Of those unsuitable for liver resection, one of the reasons cited is an inadequate future liver remnant (FLR), which puts them at risk of post-hepatectomy liver failure (PHLF). Inadequate FLR is also a major problem for patients with primary liver and biliary tract cancers who need liver resection.

PHLF is the most feared complication following major liver resection and is associated with most perioperative deaths. Mortality rates of grade A PHLF have been reported as low as 0 per cent rising to 54 per cent for grade C^1^. A low predicted FLR before liver resection is one of the key determinants in the development of PHLF. In patients with a healthy liver, a minimum FLR volume/total liver volume (TLV) of more than 25 per cent is generally considered an adequate volume, whereas in those with background liver disease an FLR/TLV of more than 40 per cent is required to produce an acceptable mortality risk^2–4^. For patients with an FLR deemed to be inadequate there are various strategies that can be used to increase the volume of the FLR to facilitate potentially curative surgery.

Portal vein embolization (PVE) is perhaps the most commonly used method to increase the FLR with a good success rate, low morbidity, and allows up to 80 per cent of patients to subsequently undergo major hepatectomy^5,6^; however, a significant proportion of patients are unable to progress to resection with the most common reason being disease progression (67 per cent) and insufficient hypertrophy of the FLR (4 per cent)^6,7^. In addition, there is evidence that PVE may stimulate cancer growth in the remnant liver due to increased post-PVE cell division with higher mitotic rates and Ki-67 proliferative index^8^.

In a meta-analysis of retrospective studies, PVE has been shown to induce comparable hypertrophy of the FLR to two stage hepatectomy with portal vein ligation^9^. More recently the associating liver partition and portal vein ligation for staged hepatectomy (ALPPS) procedure has shown very high rates of liver hypertrophy in a much shorter time frame than PVE; however, the main limitation of ALPPS is the significant postoperative morbidity and mortality rates as well as the inferior long-term survival^10–12^. It has also been reported that volumetric hypertrophy in ALPPS does not necessarily equate to function^13^, contributing to the more than expected morbidity.

In 2009, Hwang *et al.* demonstrated that sequential embolization of both the portal and hepatic veins achieved a superior FLR to PVE alone^14^. More recently simultaneous embolization of both the portal and hepatic veins, so-called liver venous deprivation (LVD) or dual vein embolization (DVE), has been shown to produce significant, rapid hypertrophy of the FLR which is comparable to ALPPS and associated with acceptable rates of postoperative morbidity^15,16^.

Several systematic reviews have been published describing the current strategies and techniques available to augment the FLR. Since then, several studies reported the results comparing PVE directly with DVE. The aim of this up-to-date systematic review and meta-analysis was to compare the outcomes of PVE alone with those undergoing DVE before major hepatectomy.

## Methods

**Fig. 1:**
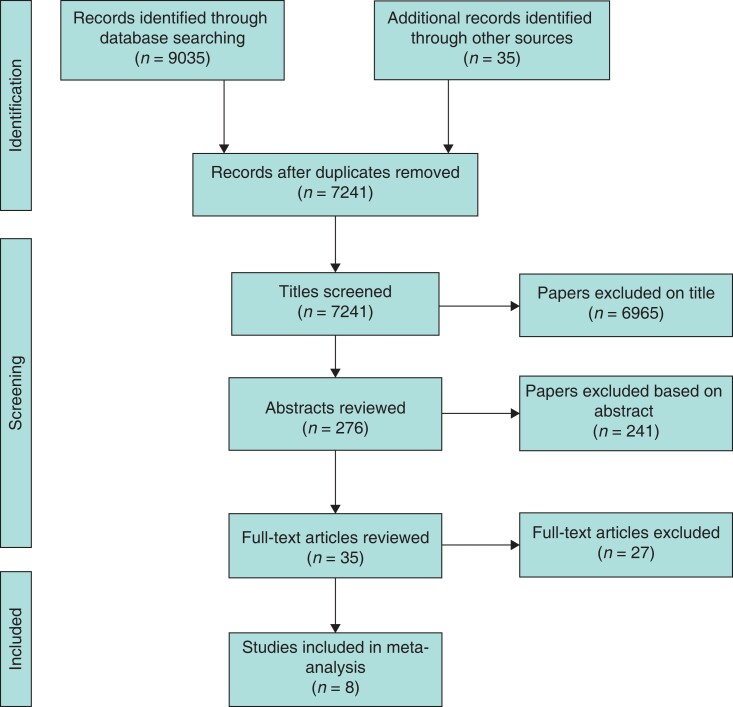
PRISMA diagram

This systematic review was conducted according to the recommendations of the PRISMA guidelines^17^. A systematic literature search was conducted up to January 2022 of MEDLINE (via PubMed), the Cochrane Library, and Embase. Abstracts and meeting proceedings were excluded but no other restrictions were applied. EU Clinical Trials Register and ClinicalTrials.gov were also searched for any ongoing trials in this field. The search strategy for this systematic review was constructed for each database by using a combination of medical subject headings (MeSH) and free-text terms as shown in the following for MEDLINE: ‘portal vein’ (MeSH) OR ‘hepatic vein’ (MeSH) AND ‘therapeutic embolisation’ (MeSH). Free-text terms included ‘dual vein embolisation’, ‘liver venous deprivation’, ‘porto-hepatic embolization’, ‘bi-embolization’, ‘hepatic vein embolization, and ‘portal vein embolization’. References from the included studies were searched to identify additional studies. Only studies that compared outcomes after simultaneous DVE with PVE before liver resection were included in the analysis. Studies comparing sequential DVE to PVE were excluded from the analysis. The PRISMA flow diagram is shown in *Fig. 1*.

## Outcome measures

As the primary intention of PVE and DVE is to increase the FLR to facilitate curative liver resection, progression to surgery was chosen as the primary outcome. Key patient-centred outcomes related to liver resection surgery were evaluated as secondary outcome measures.

## Definitions

The definition for an inadequate FLR differed from study to study and is shown in *Table S1*. PHLF was defined by the included studies according to the ISGLS definition or the ‘50–50’ criteria^18,19^. Complications were defined using the Clavien–Dindo classification with major complications classified as grade 3a or higher^20^. Perioperative mortality was defined as a patient death occurring within 90 days of surgery.

Volumetric analysis in the studies was performed using CT acquisitions of the liver. The FLR volume/transection plane was identified by a combination of experienced radiologists and surgeons. Most studies standardized the liver volume for body surface area. Two studies used specific software to calculate the volumetry.

Patient selection for either PVE or DVE varied between studies. Five studies used a time point (2016) at which they switched from predominantly using PVE to favouring DVE for managing patients with a low FLR. Two studies performed functional assessment of the FLR in addition to volumetry and favoured DVE if both assessments were low. PVE was preferred if only one of these parameters suggested an inadequate FLR.

## Statistical analysis

Quality assessment and risk of bias assessment of the included studies was based on the ROBINS-I guidelines provided by the Cochrane Collaboration^21^. Statistical analysis was performed using Review Manager version 5.3 software (Cochrane Collaboration). The OR with 95 per cent confidence interval (c.i.) was calculated for binary data and the mean difference (MD) with 95 per cent c.i. was calculated for continuous data. When median and interquartile ranges were reported their mean(s.d.) was calculated based on the methods described by Hozo *et al.*^22^. If the s.d. was not available, it was calculated as per the guidelines of the Cochrane Collaboration^23^. Random and fixed-effects models were used for each outcome^24,25^. In cases of heterogeneity, only the results of the random-effects model were reported. Heterogeneity was explored using the chi-squared test, with significance set at *P* < 0.050. Low heterogeneity was defined as an *I*^2^ value of 0 per cent to 40 per cent, moderate heterogeneity was defined as 30 per cent to 60 per cent, substantial heterogeneity as 50 per cent to 90 per cent, and considerable heterogeneity as 75 per cent to 100 per cent^26^. Forest plots were used for graphical display of the results.

## Results

Eight retrospective studies met the inclusion criteria^27–34^. All patients supplied informed consent for PVE, DVE, and surgery. Of the 523 patients included, there were 190 patients in the DVE group and 333 patients in the PVE group. The characteristics and outcomes of the included studies are shown in *Tables 1*–*3*.

**Table 1:** Characteristics of included studies

Study	Number of patients	Study interval	Age (years), mean(s.d.)	Male Sex (*n*)	BMI (mean(s.d.))	Morbidity after PVE/LVD	CRLM (*n*)	Surgery
Guiu *et al.*^27^	PVE: 22	2017–2019	66(8.5)	16	25.1(4.8)	3	17	RHH: 10 ERH: 11
	DVE: 29		62(13.25)	21	26.3(4)	6	22	RHH:13 ERH: 15
Heil *et al.*^28^	PVE: 160	2016–2019	67(2.5)	99	25.2(0.8)	25	85	RHH: 55 ERH: 50
	DVE: 39		63(3.75)	21	24.4(1.1)	6	19	RHH: 5 ERH: 29
Hocquelet *et al.*^29^	PVE: 6	2014–2018	62(3.5)	–	–	0	0*	All ERH
	DVE: 6		60(4.25)	–	–	0		
Kobayashi *et al.*^30^	PVE: 39	2010–2020	65(8.5)	19	23.8(4.1)	0	26	RHH: 19 ERH: 11
	DVE: 21		65(15)	12	23.4(4.4)	1	10	RHH: 9 ERH: 11
Laurent *et al.*^31^	PVE: 36	2016–2018	61(5.25)	26	25.5(6)	0	20	RHH: 19 ERH: 13
	DVE: 37		64(2.5)	25	25.4(7)	1	23	RHH: 10 ERH: 22
Le Roy *et al.*^32^	PVE: 41	2010–2017	63(2)	28	24(1.5)	0	–	–
	DVE: 31		66(3.75)	16	24(1)	0	18	RHH: 8 ERH: 15
Panaro *et al.*^33^	PVE: 15	2015–2017	–	–	–	0	5	All RHH
	DVE: 13		–	–	–	0	10	
Boning *et al.*^34^	PVE: 14	2015–2019	65(11)	10	26.1(4.2)	0	4	All ERH
	DVE: 14		68(10.5)	8	24.1(3.6)	2	4	

PVE, portal vein embolization; DVE, dual vein embolization; BMI, body mass index; CRLM, colorectal liver metastases; CCA, cholangiocarcinoma; RHH, right hemihepatectomy; ERH, extended right hepatectomy.

*All perihilar cholangiocarcinoma.

**Table 2:** Outcomes of included studies

Study	Number of patients	Time to resection (days), (mean(s.d.))	Failed to progress		PHLF *n*(%)	CD >IIIA *n*(%)	Perioperative mortality *n*(%)
			Total *n*(%)	Low FLR *n*(%)			
Guiu *et al.*^27^	PVE: 22	36	3 (14)	3 (100)	0 (0)	3 (16)	1 (5)
	DVE: 29	32	2 (7)	2 (100)	0 (0)	3 (11)	0 (0)
Heil *et al.*^28^	PVE: 160	41 (28–61) 41 (5.5)	51 (32)	17 (33)	27 (25)	37 (34)	17 (16)
	DVE: 39	37 (21–52) 37 (7.8)	4 (10)	1 (25)	4 (11)	9 (26)	1 (3)
Hocquelet *et al.*^29^	PVE: 6	–	1 (17)	0 (0)	2 (40)	–	2 (40)
	DVE: 6	–	2 (33)	0 (0)	2 (50)	–	0 (0)
Kobayashi *et al.*^30^	PVE: 39	35 (20–181) 35 (40.3)	9 (23)	0 (0)	–	11 (37)	0 (0)
	DVE: 21	35 (23–109) 35 (21.5)	1 (5)	0 (0)	–	7 (35)	0 (0)
Laurent *et al.*^31^	PVE: 36	44 (21–78) 44 (14.3)	4 (11)	1 (25)	7 (22)	10 (31)	1 (3)
	DVE: 37	36 (16–47) 36 (7.8)	5 (14)	0 (0)	0 (0)	6 (19)	0 (0)
Le Roy *et al.*^32^	PVE: 41	–	10 (24)	2 (20)	9 (28)	3 (9)	2 (6)
	DVE: 31	–	6 (19)	0 (0)	9 (36)	5 (20)	1 (4)
Panaro *et al.*^33^	PVE: 15	37	1 (7)	0 (0)	2 (14)	3 (21)	1 (7)
	DVE: 13	38	0 (0)	0 (0)	3 (23)	1 (8)	0 (0)
Boning *et al.*^34^	PVE: 14	–	4 (29)	0 (0)	0 (0)	–	–
	DVE: 14	–	5 (36)	0 (0)	0 (0)	–	–

Values are *n*(%) unless otherwise indicated.

FLR, future liver remnant; PHLF, post-hepatectomy liver failure; CD, Clavien–Dindo; PVE, portal vein embolization; DVE, dual vein embolization.

**Table 3:** Volumetry of included studies

Study	Group (*n*)	Pre Procedure FLR		Time to volumetry (days) median (range); mean (SD)	Post Procedure FLR		Hypertrophy (%) median (range); mean(s.d.)
		ml median (range); mean(s.d.)	% median (range); mean(s.d.)		ml median (range); mean(s.d.)	% median (range); mean(s.d.)	
Guiu *et al.*^27^	PVE: 22	542 (236–1119); 542 (221)	27.4 (13.7–47.7); 27.4 (8.5)	21	–	–	18.6 (−10.7–102.2); 18.6 (28)
	DVE: 29	484 (233–805); 484 (146)	22.6 (16.6–37.7); 22.6 (5.3)	21	–	–	52.6 (1–175.6); 52.6 (43.7)
Heil *et al.*^28^	PVE: 160	294 (233–389); 294 (26)	18.5 (15–25); 18.5 (1.7)	24 (19–37); 24 (3)	442 (342–563); 442 (37)	28 (21–37); 28 (2.7)	48 (24–69); 48 (7.5)
	DVE: 39	281 (234–352); 281 (30)	18 (16–23); 18 (1.8)	17 (13–32); 17 (4.8)	470 (382–598); 470 (54)	31 (24–39); 31 (3.75)	59 (45–79); 59 (8.5)
Hocquelet *et al.*^29^	PVE: 6	429 (391–560); 429 (42)	31 (24–33); 31 (2.2)	23.5 (15–29)	531 (500–626); 532 (31.5)	31 (24–33); 31 (2.3)	31.3 (12–40); 31.3 (7)
	DVE: 6	517 (310–828); 517 (130)	30.5 (23–33.5); 30.5 (2.6)		845 (693–960); 845 (66.8)	30.5 (23–35.5); 30.5 (3.1)	67 (29–123); 67 (23.5)
Kobayashi *et al.*^30^	PVE: 39	523 (420–659); 523 (59.8)	24 (20–33); 24 (3.3)	26 (20–33); 26 (3.3)	696 (542–819); 696 (69)	31 (25–38); 31 (3.3)	6 (1.9–9.2); 6 (1.8)
	DVE: 21	547 (435–656); 547 (55)	25 (23–31); 25 (2)	22 (17–30); 22 (3.3)	738 (662–815); 738 (38.3)	36 (31–40); 36 (2.3)	8.9 (6.7–12.8); 8.9 (30.3)
Laurent *et al.*^31^	PVE: 36	468 (253–945); 468 (173)	31 (18.3–39); 31 (5.2)	20 (25–43); 20 (4.5)	637 (326–1142); 637 (204)	39.5 (24.1–53.9); 39.5 (7.5)	29.0 (9.3–61.2); 29 (13)
	DVE: 37	387 (200–623); 387 (106)	22.9 (16.6–32.2); 22.9 (3.9)	31 (21–40); 31 (4.8)	611 (389–979); 611 (147.5)	39.9 (30.6–52.9); 39.9 (5.6)	61.2 (18–201); 61.2 (45.8)
Le Roy *et al.*^32^	PVE: 41	348 (266–547); 348 (70.3)	–	27	487 (327–612); 487 (71.3)	–	31.9+/−34
	DVE: 31	394 (262–478); 394 (54)	–	26	527 (416–662); 527 (61.5)	–	51.2+/−41.7
Panaro *et al.*^33^	PVE: 15	–	–	–	–	–	–
	DVE: 13	–	31.2+/−6.5	–	–	40.8+/−7.9	–
Boning *et al.*^34^	PVE: 14	–	–	31 (7)	–	–	–
	DVE: 14	–	–	31 (7)	–	–	–

Values are *n*(%) unless otherwise indicated.

PVE, portal vein embolization; DVE, dual vein embolization; FLR, future liver remnant.

There was no significant difference between the patients included in the DVE and PVE groups with regard to mean age (63 years *versus* 64 years, MD −0.46 (−4.03, 3.11); *Z* = 0.25; *I*^2^ = 91 per cent (*P* = 0.800)), sex distribution (61 per cent male *versus* 63 per cent male (*P* = 0.260)), BMI (24.7 kg/m^2^*versus* 24.7 kg/m^2^, MD −0.34 (−0.95, 0.27); *Z* = 1.09; *I*^2^ = 39 per cent (*P* = 0.270)). Most patients included underwent resection for colorectal liver metastases (CRLM). There was no difference between the two groups with regard to the proportion of people with CRLM (*P* = 0.990), hepatocellular carcinoma (HCC) (*P* = 0.810), intrahepatic cholangiocarcinoma (*P* = 0.670), perihilar cholangiocarcinoma (*P* = 0.190), or ‘other reasons’ for resection (*P* = 0.480).

The initial FLR volumes were comparable. The initial FLR volume (ml) was 435 ml in the DVE group *versus* 434 ml in the PVE group, MD −4.71 (−13.75, 4.32) *Z* = 0.18; *I*^2^ = 81 per cent (*P* = 0.860) and initial FLR volume (%) was 24 per cent in DVE group *versus* 26 per cent in the PVE group, MD −2.40 (−5.20, 0.41); Z = 1.68; *I*^2^ = 93 per cent (*P* = 0.090).

Post procedural morbidity was comparable (8 per cent in both groups; (*P* = 0.260)). Mean time to volumetric analysis after embolization was 23 days in the DVE group and 24 days in the PVE group, MD −0.02 (−8.25, 8.21); Z = 0.00; *I*^2^ = 98 per cent (*P* = 1.000).

**Fig. 2:**
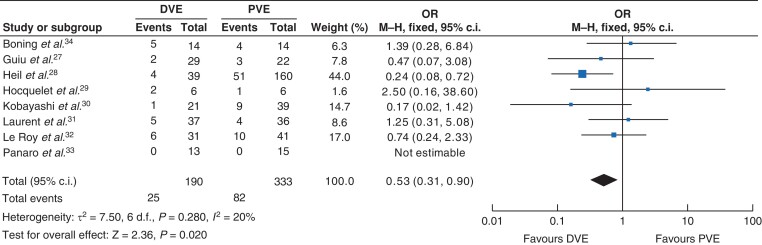
Forest plot illustrating patients failing to progress to surgery PVE, portal vein embolization; DVE, dual vein embolization.

The final FLR volume (ml and per cent) was larger in the DVE group, 638 ml *versus* 559 ml, MD 78.52 (9.95, 147.08) *Z* = 2.24; *I*^2^ 95 per cent (*P* = 0.020) and 34 per cent *versus* 32 per cent, MD 2.36 (0.17, 4.54); *Z* = 2.12; *I*^2^ 80 per cent (*P* = 0.030). The percentage increase in hypertrophy was larger in the DVE group, 66 per cent *versus* 27 per cent in the PVE group, MD 39.07 (9.09, 69.05); *Z* = 2.55; *I*^2^ = 97 per cent (*P* = 0.010). The kinetic growth rate was not reported uniformly across studies and therefore could not by analysed. The time to liver resection was shorter in the DVE group than in the PVE group, 36 days *versus* 40 days respectively, MD −4.67 (−6.97, −2.36); *Z* = 3.97; *I*^2^ = 5 per cent (*P* ≤ 0.001). Fewer patients failed to progress to surgery in the DVE group than in the PVE group, 13 per cent *versus* 25 per cent respectively, OR 0.53 (0.31, 0.90); *Z* = 2.36; *I*^2^ = 20 per cent (*P* = 0.020) (*Fig. 2*). Of the 25 patients unable to undergo liver resection in the DVE group, only 3 (12 per cent) were due to an inadequate FLR, whereas in the PVE group 23 patients of the 82 were unable to progress to resection due to FLR volume (28 per cent) (*P* = 0.270).

**Fig. 3:**
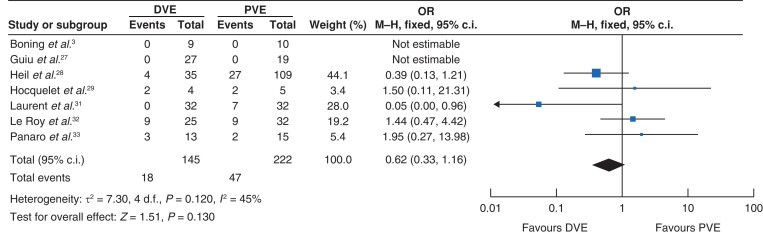
Forest plot illustrating incidence of PHLF PHLF, post-hepatectomy liver failure; PVE, portal vein embolization; DVE, dual vein embolization.

The rate of PHLF was lower in the DVE group, 13 per cent *versus* 22 per cent respectively, OR 0.62 (0.33, 1.16); *Z* = 1.51; *I*^2^ = 45 per cent (*P* = 0.130) (*Fig. 3*). The included studies did not report the grades of PHLF to further assess the difference in the incidence of the grades of PHLF.

**Fig. 4:**
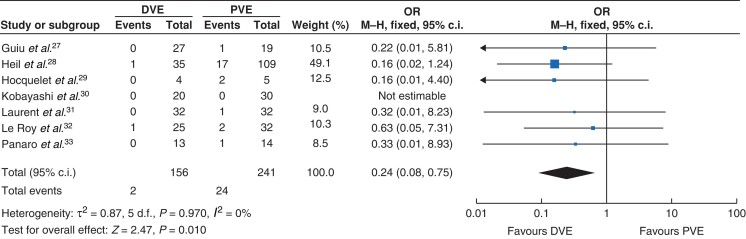
Forest plot illustrating postoperative mortality PVE, portal vein embolization; DVE, dual vein embolization.

There was no statistical difference in major complications between the two groups, 20 per cent in the DVE group *versus* 28 per cent in the PVE group, OR 0.76 (0.46, 1.25); *Z* = 1.08; *I*^2^ = 0 per cent (*P* = 0.280). Perioperative mortality was lower in the DVE group 1 per cent *versus* 10 per cent OR 0.24 (0.08, 0.75); *Z* = 2.47; *I*^2^ = 0 per cent (*P* = 0.010); *Fig. 4*.

## Discussion

The present review has demonstrated that DVE is associated with a greater degree of hypertrophy of the FLR with a higher proportion of patients progressing to surgery than PVE alone. In addition, DVE seems to be a safe procedure with equivalent procedure-related morbidity. While not statistically significant, rates of PHLF were 40 per cent lower following major hepatectomy in patients who had undergone DVE, and major complications were 29 per cent lower, which seems to translate into a lower perioperative mortality with DVE probably due to the improved FLR.

The most common complication following DVE was a post-embolization syndrome (9 of 16) characterized by abdominal pain and fever, managed conservatively, with other more serious complications including a haemoperitoneum and haemobilia both managed conservatively, two patients with a perihepatic haematoma were managed conservatively with one death post-procedure due to sepsis from infected tumour necrosis. There were two cases of non-target embolization. There were no cases of technical failure of either PVE or DVE. Complication rates with DVE and PVE were comparable between the studies.

This study has also demonstrated that fewer patients undergoing DVE fail to progress to liver resection surgery than following PVE alone. Of the 20 patients unable to undergo liver resection in the DVE group, only three (15 per cent) were due to an inadequate FLR, whereas in the PVE group 23 patients of the 79 were unable to progress to resection due to FLR volume (29 per cent). This, however, did not reach statistical significance (*P* = 0.270). Most of the remaining patients in both groups developed disease progression either on imaging or at time of surgery that precluded proceeding with liver resection. No studies reported any longer-term follow-up or survival analysis to allow calculation of a survival benefit associated with DVE despite the increased rate of patients progressing to liver resection. Data from the LIGRO trial, suggest the survival benefit of those who had completion hepatectomy (40 of 50 patients, median survival of 32 months) compared with those who did not (10 of 50 patients, 13 months) following PVE or ligation. Based on these data a survival advantage may exist with DVE over PVE and needs investigation in future studies^35^.

While this study has demonstrated a greater increase in FLR volume with DVE, PHLF was encountered in 13 per cent of patients proceeding to resection and it must be remembered that volume does not necessarily equate to function, which is similar to the ALPPS cohort. Dynamic 99mTc-mebrofenin hepatobiliary scintigraphy with single-photon emission CT is one method that has been used to quantitively assess liver, and FLR function^36^. Guiu *et al.* investigated the impact of PVE and DVE using 99mTc-mebrofenin SPECT-CT measuring function and volume at day 7, 14, and 21 after the procedure. FLR function and volume was significantly greater at all time points with DVE as opposed to PVE alone^27^. The HYPER-LIV01 trial (registration number: NCT03841305 (http://www.clinicaltrials.gov)) is a multicentre French trial that is currently recruiting and will compare patients with operable CRLM with an FLR less than 30 per cent randomizing to either PVE or DVE. The FLR will be assessed not only for change in volume but also function using 99mTc-mebrofenin SPECT-CT and will add considerably to the evidence base for DVE^37^. In addition to the HYPER-LIV01 trial, the Maastricht Group are also running the prospective DRAGON-1 study (DRAGON-1 –Training, Accreditation, Implementation and Safety Evaluation of Combined PVE/HVE (registration number: NCT04272931 (http://www.clinicaltrials.gov)) to assess safety and feasibility of DVE in patients with CRLM as a precursor to a randomized clinical trial comparing PVE and DVE (DRAGON-2) with the results awaited.

The present study has several limitations. There is a clear selection bias with regard to which patients received either PVE or DVE. Four studies primarily favoured DVE from 2016 onwards as familiarity with the technique grew and its safety was demonstrated; however, two studies used DVE in patients with low volume and functional assessment, whereas PVE was used if only one of these parameters was low. This would mean that patients at higher risk of postoperative morbidity and PHLF therefore preferentially received DVE. Despite this, morbidity, and PHLF rates were lower with DVE. Although the percentage increase in FLR is unlikely to be influenced by segment-4 portal embolization, it can clearly influence the rates of progression to completion surgery in those requiring more extended hepatectomy. The reasons for the selection of patients for additional middle hepatic vein or segment-4 portal embolization are not clearly defined within the studies. Another factor is the variation between studies with regard to what constitutes an adequate FLR, although this is unlikely to influence the percentage increase in FLR. In addition, the definition for PHLF used between studies differed although all studies adopted either the ‘50–50’ criteria or ISGLS definition^18,19^. Factors that can impact on the hypertrophy of FLR such as presence of background fibrosis or cirrhosis, extent of chemotherapy were not matched between the groups. All patients in this study also underwent PVE with N-butyl cyanoacrylate glue with varied hypertrophy rates. Some studies have demonstrated that the addition of a central vascular plug or coil in PVE alone is associated with increased hypertrophy rates which may be related to revascularization of the portal vein^38^. Therefore, the technique for PVE used in these studies may be associated with an inferior hypertrophy than more current techniques; however, the technique for PVE was the same for the DVE and PVE groups of each study included.

Given the quality and limitations of the literature comparing PVE with DVE it is difficult to draw firm conclusions about the superiority of DVE over PVE. Studies evaluating DVE are small, retrospective, and have no longer-term follow-up. As such, DVE is still considered by many to be an evolving technique. In addition, the randomized studies comparing DVE to PVE that are currently recruiting using surrogate endpoints such as percentage change in FLR with only short-term follow-up available. Nevertheless, until prospective and controlled studies are available, this study represents the best available evidence at present.

## Conclusion

DVE seems to be a safe technique which produces a greater degree of FLR hypertrophy when compared with PVE alone. This translates into more patients undergoing surgical resection and lower rates of PHLF, major complications, and perioperative mortality. High-quality studies are needed to confirm these findings.

## Supplementary Material

zrac131_Supplementary_Data

## Data Availability

The datasets generated during and/or analysed during the current study are available from the corresponding author on reasonable request.
