# Supplementary material for: Portal vein embolization versus dual vein embolization for management of the future liver remnant in patients undergoing major hepatectomy: meta-analysis
Source: BJS Open. 2022 Nov 18;6(6):zrac131. doi: 10.1093/bjsopen/zrac131 (PMC9673134; doi:10.1093/bjsopen/zrac131)
Supplement: zrac131_Supplementary_Data [file zrac131_supplementary_data.docx]

Table S1: Study Definitions

|  | **FLR Definition** |  | **PHLF** | **Procedure** | **Seg 4 and MHV Embolisation** | **Risk of Bias*** |
| --- | --- | --- | --- | --- | --- | --- |
| Guiu et al ^25^ | <30% of baseline (-tumour volume) | Excluded cirrhosis, hilar cholangio, 2 stage hepatectomy | ’50-50’ criteria | PVE – R portal access, n-BCA + ethiodized oil. Segment 4 not embolised | RPV + P4 - NS | Moderate |
|  |  |  |  | Hepatic Vein – RHV +/- accessory RHV +/- MHV  Oversized Amplatzer Vascular Plug + nBCA + ethiodized oil | RPV + P4 – NS  MHV - NS |  |
| Heil et al ^26^ | <30% FLR | All tumour types  (Excl ALPPS and PVL) | ISGLS | PVE - Majority - R portal, n-BCA + ethiodized oil | RPV + P4 -NS | Moderate |
|  |  |  |  | Hepatic Vein Variety – RHV +/- accessory RHV +/- MHV  Amplatzer +/- nBCA/ethiodized oik or coils | RPV + P4 - NS  MHV - 11 |  |
| Hocquelet et al ^27^ | <40% (all hilar cholangio) | All had simultaneous biliary drainage | ’50-50’ criteria | PVE – L portal access, n-BCA + ethiodized oil | RPV + P4 – 0 | Moderate |
|  |  |  |  | Hepatic vein – transjugular, RHV +/- accessory RHV. No MHV. Oversized Amplatzer plug | RPV + P4 - 0  MHV - 0 |  |
| Kobayashi et al ^28^ | <30% normal liver, <35% impaired liver function/post chemo |  | Not defined | PVE – L portal access, n-BCA and ethiodized oil | RPV + P4 - 3 | Moderate |
|  |  |  |  | Hepatic vein – transjugular, RHV +/- MHV, multiple Amplatzer plugs | RPV + P4 - 1  MHV - 0 |  |
| Laurent et al ^29^ | Normal <25%  Disease <35% | Fibrosis/cirrhosis excluded | ISGLS | PVE – R or L access, no segment 4, n-BCA and iodized oil | RPV + P4 - 0 | Moderate |
|  |  |  |  | Hepatic Vein – RHV +/- aRHV +/- MHV, Amplatzer plug + n-BCA and iodized oil | RPV + P4 - 0  MHV - 0 |  |
| Le Roy et al ^30^ | Normal <25%  Diseased <40% | TACE excluded | ’50-50’ and ISGLS | PVE – L portal access, n-BCA + iodized oil | RPV + P4 - NS | Moderate |
|  |  |  |  | Transjugular – oversized plug, no coils | RPV + P4 - NS  MHV - NS |  |
| Panaro et al ^31^ | Normal <25-30%  Diseased <35-40% | All patient biopsied to evaluate parenchyma  Only standard right hepatectomy | ISGLS | PVE – R transhepatic, n-BCA + iodized oil | RPV + P4 - NS | Moderate |
|  |  |  |  | Hepatic veins – RHV +/- aRHV, oversized amplatzer plug +n-BCA + iodized oil | RPV + P4 - NS  MHV - NS |  |
| Boning et al ^32^ | NS | Must have Plt >50,000/nl, INR <1.5, PTT <50 s | NS |  | RPV + P4 - NS | Moderate |
|  |  |  |  | Hepatic veins – 30% oversize Amplatzer plug, n-BCA and ethiodized oil | MHV - NS |  |

* As assessed by ROBINS-I, FLR – future liver remnant, ALPPS – Associating liver partion and portal vein ligation for staged hepatectomy, PVL – portal vein ligation, TACE – transarterial chemoembolization, ISGLS – International Study Group for Liver Surgery, PVE – portal vein embolization, n-BCA – n-butyl-cyanoacrylate, RHV – right hepatic vein, MHV – middle hepatic vein, NS – not stated
